# Dynamic intravital imaging reveals reactive vessel-associated microglia play a protective role in cerebral malaria coagulopathy

**DOI:** 10.1038/s41598-023-43208-5

**Published:** 2023-11-09

**Authors:** Olivia D. Solomon, Paula Villarreal, Nadia D. Domingo, Lorenzo Ochoa, Difernando Vanegas, Sandra M. Cardona, Astrid E. Cardona, Robin Stephens, Gracie Vargas

**Affiliations:** 1 The Institute for Translational Sciences, University of Texas Medical Branch, Galveston, TX 77555 USA; 2 Biomedical Engineering and Imaging Sciences Group, University of Texas Medical Branch, Galveston, TX 77555 USA; 3 Department of Neurobiology, University of Texas Medical Branch, Galveston, TX 77555 USA; 4 Center for Immunity and Inflammation, Rutgers New Jersey Medical School, Newark, NJ 07103 USA; 5 Department of Internal Medicine, Division of Infectious Diseases, University of Texas Medical Branch, Galveston, TX 77555 USA; 6 Department of Molecular Microbiology and Immunology, University of Texas at San Antonio, San Antonio, TX 78249 USA; 7 Department of Pharmacology, Physiology and Neuroscience, Rutgers New Jersey Medical School, Newark, NJ 07103 USA; 8 Department of Microbiology and Immunology, University of Texas Medical Branch, Galveston, TX 77555 USA

**Keywords:** Glial biology, Neuroimmunology, Infectious diseases

## Abstract

Vascular congestion and coagulopathy have been shown to play a role in human and experimental cerebral malaria (eCM), but little is known about the role of microglia, or microglia-vascular interactions and hypercoagulation during disease progression in this fatal infection. Recent studies show microglia bind to fibrinogen, a glycoprotein involved in thrombosis. An eCM model of *Plasmodium chabaudi* infection in mice deficient in the regulatory cytokine IL-10 manifests neuropathology, including hypercoagulation with extensive fibrin(ogen) deposition and neuroinflammation. Intravital microscopy and immunofluorescence are applied to elucidate the role of microglia in eCM. Results show microgliosis and coagulopathy occur early in disease at 3 dpi (day post-infection), and both are exacerbated as disease progresses to 7dpi. Vessel associated microglia increase significantly at 7 dpi, and the expression of the microglial chemoattractant CCL5 (RANTES) is increased versus uninfected and localized with fibrin(ogen) in vessels. PLX3397 microglia depletion resulted in rapid behavioral decline, severe hypothermia, and greater increase in vascular coagulopathy. This study suggests that microglia play a prominent role in controlling infection-initiated coagulopathy and supports a model in which microglia play a protective role in cerebral malaria by migrating to and patrolling the cerebral vasculature, potentially regulating degree of coagulation during systemic inflammation.

## Introduction

Malaria is a life-threatening disease afflicting approximately 247 million individuals worldwide each year^1^. Infection with *Plasmodium falciparum*, the deadliest malaria parasite, caused approximately 619,000 deaths in 2021, with children bearing the greatest burden. Cerebral malaria (CM), a lethal neurological outcome of systemic *P. falciparum* infection, is a leading cause of malaria mortality accounting for two-thirds of *P. falciparum* malaria deaths^1^. This encephalopathy results in neurological symptoms including seizures and impairment of consciousness^2^, along with a high level of asexual forms of the parasite in peripheral blood. Although edema is implicated in final cause of death and up to 20% of hospitalized patients succumb to the disease, there is a lack of understanding of the lethal cerebral pathogenesis. Additionally, 15–20% of CM survivors are left with long term neurological sequelae^3^ that include transient ataxia, cortical blindness, impaired cognition, and language and behavioral problems indicating multiple regions of the brain may be impacted, though symptoms differ between patients^4,5^.

Dysregulation of inflammation and coagulation pathways are reported to occur in CM and implicated in its pathogenesis. Clinically, a procoagulant state has been suggested to occur in pediatric CM with elevated plasma levels of fibrin degradation products in children with CM compared to those with mild malaria^6,7^. Notably, 17% of all CM patients have all the symptoms of disseminated intravascular coagulopathy, and this correlates to increased mortality^8^. Indicators of dysregulated coagulation and endothelial cell activation in *P. falciparum* infection include thrombocytopenia, elevated plasma levels of D-dimers, thrombin-antithrombin III complexes (TAT) fibrinopeptide A, and reduced antithrombin III^8,9^. Post-mortem brain samples from CM patients show hallmark histological features of vessel leakage and vessel occlusions including cerebral thrombi with and without parasite enrichment, indicating parasite sequestration on the vascular endothelium^10^. Clots were observed with reduced endothelial expression of anticoagulant protein C and platelet accumulation in cerebral vessels^10,11^. Also, postmortem brain tissue from pediatric *P. falciparum* CM cases show positive staining for tissue factor in the vascular endothelium^12^. These findings indicate a role of coagulation in CM, though there is a need to understand the role of coagulation as it relates to disease progression and in relation to neuroinflammation.

Early glial activation markers in blood vessels with sequestered infected red blood cells in autopsy brains of CM patients indicate microglial involvement in human CM^13^. However, the role of microglia and impact of microglial activation in CM is not known. Microglia are the primary immune effector cells of the central nervous system responsible for maintaining homeostasis. Homeostatic microglia survey their environment for insult. Microglial activation may occur by a variety of stimuli, including in pro-inflammatory cytokines, and has been shown to occur in eCM^14,15^. Reactive microglia can respond with phagocytosis and cytokine production. Microglia display a wide spectrum of activation phenotypes to act in beneficial (e.g. synaptic pruning) or damaging (e.g. release of proinflammatory cytokines) manners.

As in humans, experimental models (eCM) also present coagulopathy and microgliosis. Mice deficient in the regulatory cytokine IL-10 develop a hyperinflammatory response and cerebral pathology to the mildly pathogenic parasite *P. chabaudi*, including significantly increased fibrin(ogen) deposition in brains of infected animal with the 60-100% fatality, often studied in advanced disease^15,16^. Occlusive thrombi occur in cerebral vessels in multiple areas of the brain, with leukocytes contained within some congested vessels, an indication of microgliosis and astrogliosis in advanced disease^15^. Treatment with either low molecular weight heparin (LMWH, Enoxaparin) or neutralizing antibody to the pro-inflammatory cytokine (TNF) resulted in reduced thrombosis, and attenuation of gliosis, as well as increased mouse survival and improved behavior symptoms, suggesting the possibility of intersecting roles for coagulation and neuroinflammation in eCM^15^. The pro-inflammatory cytokine TNF, upregulated in human and murine CM can also activate coagulation via tissue factor, an initiator of the extrinsic coagulation cascade and may inhibit protein C activation^17^.

The current study was carried out to investigate the roles of microglia in relation to vascular dysregulation, specifically coagulopathy, with studies conducted in a *P. chabaudi model* in IL-10 KO CX3CR1-GFP CCR2-RFP mice. Additional limited, complementary studies in a *Plasmodium berghi ANKA* (PbA) model were conducted to demonstrate similarity in microglial responses observed following *P. chabaudi infection* with this established and commonly studied eCM model*.* Real-time dynamics of microglial responses and their associations with the vasculature, not previously explored in eCM, were investigated using intravital two-photon microscopy and fluorescent fibrin(ogen) in both eCM models, which demonstrated vessel occlusions, microglial activation, and microglial association with cerebral vessels. In *P. chabaudi* eCM, early and late timepoints were studied to determine the kinetics of microglial activation and vascular congestion with fibrin(ogen) deposits. We report that microglia are activated early in infection and are increasingly associated with the vasculature. Microglial colocalization near thrombosed vessels corresponds to the increased expression of the chemoattractant CCL5. Strikingly, depletion of microglia with PLX3397 treatment resulted in increased disease severity and coagulation, suggesting microglia take on a protective role in eCM and ameliorate the impact of coagulopathy.

## Methods

### Mice

C57BL/6J (WT,B6) and B6.129P2-Il10^tm1Cgn^/J (IL-10 KO), CX3CR1^GFP^, IL-10 KO CX3CR1^GFP/-^ CCR2^RFP/-^ mice used in these studies were bred at the University of Texas Medical Branch Animal Resource Center. The CX3CR1^GFP/-^ CCR2^RFP/-^ mice were crossed with IL10 KO mice to create the double transgenic IL-10 KO CX3CR1^GFP/-^ CCR2^RFP/-^ in which microglia in IL-10 KO mice may be identified by green fluorescent protein (GFP) expression and CCR2 monocytes by red fluorescent protein (RFP) expression. Experimental mice include males and females ages 6 to 12 weeks weighing at minimum 16 g at time of infection with *P. chabaudi *(*IL-10 KO,* IL-10 KO CX3CR1^GFP/-^ CCR2^RFP/-^) *or P. berghei ANKA* (C57BL/6J (WT) & CX3CR1^GFP^). Mice were kept in pathogen-free housing with ad libitum access to water and food. The study was approved by the Institutional Animal Care and Use Committee (IACUC 1904044A). University of Texas Medical Branch’s ARC facilities operate in compliance with the USDA Animal Welfare Act, the Guide for the Care and Use of Laboratory Animals, under OLAW accreditation, and IACUC-approved protocols; animals were cared for according to the Guide for the Care and Use of Laboratory Animals.

### Parasite and infection

Frozen stocks of *P. chabaudi chabaudi* (AS) (originally from Jean Langhorne, Crick Institute, London, UK) *and P. berghei ANKA* (from BEI Resources Repository, NIH) infected red blood cells (iRBCs), kept in liquid nitrogen, were quickly thawed and injected into WT (B6) mice intraperitoneally (i.p.). Parasitized blood was collected from infected mice before the peak of infection and diluted in Krebs–Ringer bicarbonate buffer (Sigma-Aldrich, St. Louis, MO) and normal Saline (Sigma-Aldrich, St. Louis, MO) by standard protocol (Slade, 1989). For *P. chabaudi* infection, experimental mice (IL-10 KO or IL-10 KO CX3CR1^GFP/-^ CCR2^RFP/-^) were given 10^5^ iRBCs i.p. in 200 µL. Uninfected matched-model mice were used for controls. In all cases, peripheral parasitemia was confirmed by counting parasitized RBCs on a light microscope in thin blood smears collected at regular intervals (5–7 dpi) and stained with Diff-Quik (Fisher Scientific, Hampton, NH).

### Monitoring body temperature and weight

Mice were assessed daily starting a 5 dpi during infection periods for internal body temperature using a stainless steel rectal probe and BIO-TK8851 digital rodent model thermometer (Braintree Scientific, Braintree, MA). The probe was sanitized after each mouse with CaviCide and allowed to dry before reuse. During these daily assessments, weights were recorded using an OHAUS Scout Pro SP601 portable balance (OHAUS, Parsippany, NJ).

### Animal behavior

Starting on 6 dpi, mice were assessed daily for behavior using an abbreviated version of the SmithKline Beechman, Harwell, Imperial College, Royal London Hospital Phenotype Assessment (SHIRPA) protocol^18^. This abbreviated behavioral assessment developed by Wilson et al., focuses on metrics for general health, sensory function, baseline behavior, and neurological reflexes of the SHIRPA protocol. Higher behavioral scores are given to mice who exhibit higher functional ability versus mice who score lower. The abbreviated-SHIRPA involves nine semi-quantitative assessments that include: body position, spontaneous activity, palpebral closure, gait, tail elevation, touch escape, grip strength, heart rate, and righting reflex, as reported in Wilson et al.^15^. All control mice used include un-infected IL10 KO CX3CR1^GFP/-^CCR2^RFP/-^, which received scores ranging from 21–22. Mice determined to have cerebral malaria typically have a score less than 16^15^. Animals were excluded from the study if behavior scores did not indicate cerebral malaria and/or no presence of parasite in blood smears.

### Intravital dyes

Intravascular fibrinogen-conjugated florescent labels and vascular contrast dyes were employed for intravital visualization of fibrinogen and cerebral vessels, respectively. Alexa dye-conjugated fibrinogen (1.5 mg/mL; Alexa 488, 594, 647, ThermoFisher Scientific, Waltham, MA) was prepared as per previously published protocols^19,20^. Briefly, Alexa dye conjugated fibrinogen was prepared using 3.333 mL of sterile fresh sodium bicarbonate solution added to a 5 mg vial of the Alexa conjugated fibrinogen and vortexed until completely dissolved. A volume of 100 µl of prepared Alexa conjugated fibrinogen was administered intravenously (i.v.) at both 48 h and 24 h prior to intravital imaging. A solution of 2% Evans Blue was administered via intraperitoneal injection (4 ml/kg) for visualization of cerebral vessels by intravital microscopy; freshly prepared with aseptic phosphate buffered saline solution, based on prior studies^21^. A combination of fluorescent probes is utilized during intravital imaging experiments. In some cases, we used Alexa488 conjugated fibrinogen to visualize fibrin(ogen) + coagula within the vasculature delineated by Evans Blue. In other experiments where fibrinogen-microglial interaction is investigated to prevent spectral overlap, Alexa647 conjugated fibrinogen was utilized and no vascular probe was applied. In this work the term fibrin(ogen) will be used when fibrin and fibrinogen are indistinguishable, as conversion of fibrinogen to fibrin in the Alexa dye-conjugated fibrinogen maintains fluorescence.

### Thin skull cranial preparation

For intravital imaging, experimental mice were anesthetized with a mixture of ketamine/dexmedetomidine (0.1 ml/10 g) and kept on a heating pad with tail and toe pinch responses monitored. The head was immobilized with a stereotactic device (Stereotaxic 520, Steoelting: Wood Dale, IL) during the thinning procedure. Ophthalmic ointment (NDC 11695-6832-1, Covetrus: Dublin, OH) was placed on the eyes of the mouse to prevent drying out. The surgical area was cleaned with 70% alcohol and then wiped using room temperature PBS. A hair depilatory (Nair, Ewing, NJ) was applied, and a midline section cut was lightly made on the skin of the head. An imaging headpost composed of Zinc, with a diameter of 1/2 inch (229489, Home Depot; Atlanta, GA) was applied to the head of the mouse using Cyanoacrylate (EM-02, Starbond: Los Angeles, CA) to maintain liquid tension during intravital imaging. Fascia was scraped away to completely reveal the skull. Thin skull cranial windows were performed just below the coronal suture & bregma and parallel to the sagittal suture. A high-speed dental drill (NC9010016, Braintree: Braintree, MA) with a round carbide bur (Meisinger: Centennial, CO) was used to gently make circular motions to remove a portion of the spongy bone until pial blood vessels can be visualized under a stereotactic microscope (SMZ445, Nikon: Brighton, MI) and the remaining bone shows flexibility to gentle pressure from the drill tip. PBS or ACSF (597316, Harvard Apparatus: Holliston, MA), previously warmed in an incubator, were frequently reapplied during pauses in drilling to prevent drying, overheating, and induction of inflammation, and to remove debris. If bleeding occurred due to rupture of vessels in the spongy bone, gel foam (09-0315-08, Pfizer: New York, NY) or bone wax (W31G, Ethicon: Cincinnati, OH) was applied to impacted areas and fresh PBS applied. Before placing the mouse under the objective of the upright microscope, the surgical area was completely cleaned, and fresh PBS applied to aid visualization under the microscope.

### Intravital two-photon fluorescence excitation microscopy

To visualize cortical vasculature and other fluorescent structures, intravital microscopy (IVM) was performed using two-photon fluorescence excitation microscopy (TPEM). TPEM was performed using an Ultima IV upright multiphoton microscope (Bruker, Middleton, WI) with a Mai Tai ultra-fast femtosecond laser as an illumination source (Spectra Physics, Santa Clara, CA) tuned to 800nm for florescence excitation. Upon completion of thin skull cranial window technique, the anesthetized mouse was placed prone on a heating pad is placed on a custom-made stage under a 40 × 0.8 N.A. water immersion objective (NIR Apo, 0.80W, DIC N2, WD 3.5) focused on the superficial cortex and providing a field-of-view of 321.38 X 321.38 $\mu$m. Custom cubes for two channel acquisitions were used. In acquisitions targeting GFP &/or Alexa488 together with RFP used the following filter 1: 604 /45nm bandwidth, filter 2: of 525/70 nm bandwidth, and a dichroic mirror (575 nm). For the acquisition of Alexa 647 an additional cube that included the bandwidth of 660/40m (dichroic, 585 nm) was used for the red channel. Data taken in these studies include time series (T-series) to assess dynamic changes over a period of time and depth series (Z-series) for three-dimensional spatial reconstructions. Image stacks were taken at approximately 4–6 sites per mouse. T-series acquired were taken with a 2.969 ms interval and a frame size of 512 × 512. Time series acquired totaled to either 15, 35, or 45 min depending on the number of frame iterations acquired: 250, 500, or 1000 iterations, respectively. Z- series range in depth from 50 to 200 µm with a step size of 1.0 µm and frame size of 1024 × 1024.

Ex vivo imaging was performed using MicroBrightField widefield fluorescence (objective: Plan-Apochromat 20X NA 0.8) and Zeiss Inverted LSM 800 confocal (objective: LD LCI Plan-Apochromat 20X NA 1.2 water immersion) microscopes.

### Antibodies

Primary antibodies included rabbit anti-fibrinogen (A0080, Dako, Glostrop, Denmark), anti-CCL5 (SC-365826, Santa Cruz Biotechnology, Dallas, TX), anti-Iba-1 (019-19741, Fujifilm Labchem Wako, Osaka, Japan), and anti-TMEM119 (ab209064, ABCAM, Cambridge, UK). Secondary antibodies used include Alexa Fluor405 goat anti-rabbit IgG (A31556 Invitrogen, Eugene, OR), Alexa 568 goat anti-rabbit IgG (A11012, Invitrogen Eugene, OR), Alexa 594 goat anti-rabbit IgG (A11012, Invitrogen Eugene, OR), Cy3 goat anti-rabbit (A10520 Life Technologies, Eugene, OR), and Dylight 594 (DL-1177, Vector, Burligame, CA) was used to visualize the vasculature. It is noted that anti-fibrinogen antibody reacts with fibrinogen, fibrin and the fibrinogen fragments D and E, typical of such antibodies, thus, “fibrino(gen)” is used in staining results.

### Immunofluorescence

For ex vivo studies, right atrial perfusion was performed using cold PBS and 4% paraformaldehyde (PFA). Extracted tissues were then fixed overnight in 4% PFA at 4 °C. Fixed whole brains were hemisected and cryoprotected by incubation in 30% sucrose for 3 days, hemisected brains placed in 15ml conical tubes in 4 °C. For cryosectioning, tissues were embedded in Optimal Cutting Temperature Compound (OCT 4585, Fisher Healthcare, Houston, TX) until hardened in a  − 80 freezer (~ 3 h). Thirty-micron sagittal sections were cut using a cryotome and allowed to free float in a 12 well flat bottom cell culture plate (3513, Corning Inc) with 1000 ml of fresh PBS to remove OCT medium. Tissues were blocked in 5% goat serum diluted from a 10% serum (Goat Serum 500,622, Life Technologies, Frederick, MD) in PBS with Triton-X100 for 1 h at room temperature. The primary antibody was incubated overnight with gentle agitation at room temperature or 4 °C as per manufacturer recommendation, specified below. Brain sections were then washed three times with PBS/Triton-X100 (PBST) in 15 min intervals with gentle agitation. The secondary antibody was incubated overnight with gentle agitation at room temperature. The sections were then washed with PBS and mounted onto glass slides with mounting media (0.5% DMSO, 50% TDE, & 49.5% PBS).

### Image analysis

Image files were acquired in 12-bit Tiff format, saved in 16-bit Tiff, and processed for various variables using Fiji-ImageJ (version 2.1.0 Java 1.8.0, NIH) and/or Imaris (Bitplane 9.7). For analysis and quantification, 8-bit Tiff images were imported and processed using Fiji-ImageJ by applying a background subtraction with a rolling ball radius of 25 pixels, median filter with a radius of 2 pixels, and despeckled to remove any nonspecific noise. Fiji-ImageJ software was used to complete most measurements as described below for analysis of IVM images. T-series data was also evaluated using Fiji-ImageJ to create movies to assess real time dynamics of microglia and vascular dysfunction. Imaris was utilized to create 3-dimensional renderings of depth stacks acquired from experiments to further assess spatial distribution, microglia, and morphology^22^.

### Analysis of IVM images and immunofluorescence markers

Microglia in imaging fields were subjectively based on morphology and were grouped into 4 categories, ramified/surveying, hypertrophic, rod, and ameboid. Percentage of each type were calculated across fields per mouse (mean: 25 microglia per field).

Soma diameter analysis was quantified manually by using the line tool using Fiji-ImageJ on calibrated images. Measurements were taken from depth series and using ROIs to measure the longest diameter & shortest diameter, averaged to determine the maximal diameter. A total mean measure was determined for each field and averaged across all animals for each group.

An analysis of Vessel associated microglia (VAM) was performed by enumerating the number of microglia soma in direct contact with a vessel. Assessments were made in intravital microscopy depth stack images, with 3–4 fields per mouse. The percentage of VAMS is as follows: # of CX3CR1^GFP^ + soma at vessel/ total # of CX3CCR1^GFP^ + microglia. GFP + meningeal macrophages identified by morphology and depth and were excluded from analysis. Microglia were identified based on GFP + signal and morphology.

Statistical association between microglia based on morphology type with cerebral vessels was determined using the chi-square test of independence, and a 2-way contingency table created to visually relay degree of association. The formula for this statical test is as follows:

$$\sum {{\text{ri}} = 1\sum {{\text{cj}} = 10{\text{ij}} - {\text{EijEij}}} }$$

This statistical test is represented as an association plot exhibiting the standardized residuals and the square root of the expected frequency. Graphical representation exhibits the nature of dependency between the variables and the relative contribution of each event in the association matrix to the total chi-square score^23^.

Microglia engulfment of florescent fibrin(ogen) was determined by assessing colocalization of fluorescent fibrin(ogen) signal and microglia (CX3CR1^GFP^ +) and calculating # of microglia localized with fibrin(ogen)/ total # of microglia)*100.

Sholl analysis and transformation index was used to assess IBA-1 immunofluorescence of microglia activation based on morphology using Fiji-ImageJ software. The first shell in Sholl analysis was set 10µm outside of the soma to exclude the soma from measurements with parameters set to linear with a step size of 5µm. Transformation Index was calculated by as Perimeter^2^/4pArea.

### Statistics

Where indicated in figure legend, groups were compared either by Welch’s t test or one-way ANOVA followed by post hoc Holm test. Statistical tests were chosen based on the number of groups and to account for unequal variances. Post hoc Holm correction was used for one-way ANOVA to account for Type 1 errors. Statistical analyses were performed in Prism (GraphPad, La Jolla, CA), while chi square analysis was performed in R studio (R Foundation, Vienna, Austria). Statistical significance is indicated as *p = 0.05, ** *p* = 0.01, ***P = 0.001, ****P = 0.0001 . Error bars represent standard deviation. The following sample sizes were used in these studies, n = 3–5 mice/per group with 3–5 sites assessed per mouse, each data point in graphs represents the average value per animal after analysis.

### ARRIVE

The study reported is in accordance with ARRIVE guidelines.

## Results

### *P. chabaudi* infection of IL-10 KO mice causes microglial association with coagulated cerebral vessels

**Figure 1:**
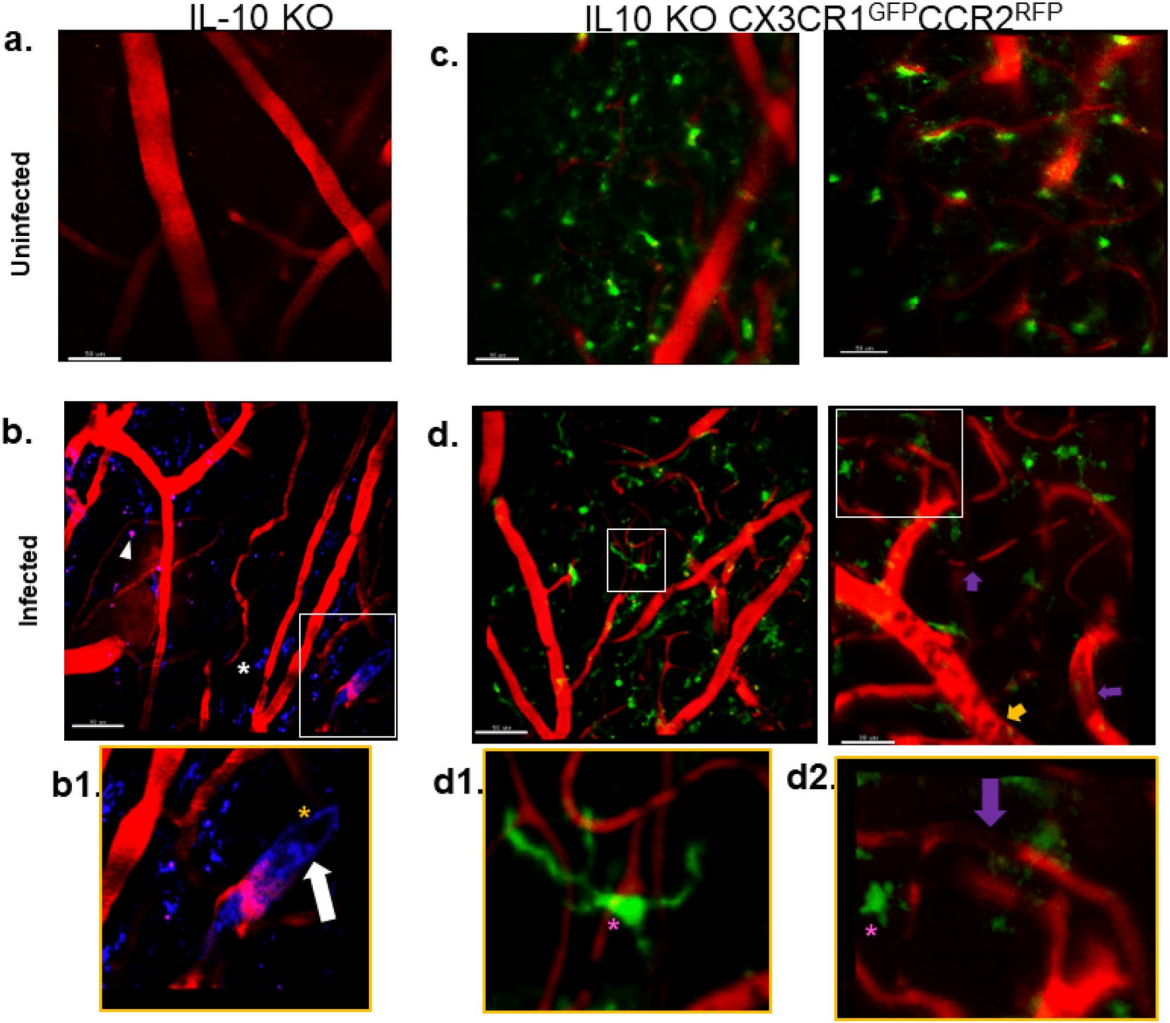
Microglial-vascular interaction and coagulation in hyperinflammatory eCM revealed via intravital microscopy. IVM volumetric projection of cerebral vessels of the cortex in (**a**) uninfected or (**b**) infected IL-10 KO with Evans blue (EB, red) and fluorescent fibrin(ogen) (blue) on day 7 of infection showing coagulation (inset). Extravascular fibrin(ogen) deposits also visible as puncta (white asterisk) and larger globules in panel and inset (**b1**) Solid arrow shows fully occluding thrombus and non-fluorescent vascular congestion (gold asterisk inset and panel. Darkened regions in vessels denoted by gold asterisk exhibiting congestion. (**c**) Ramified Microglia (Green) in cortex in uninfected IL-10 KO CX3CR1^GFP^ CCR2^RFP^. (**d**) Activated microglia (hypertrophic) interacting with vasculature in *P. chabaudi*-infected IL-10 KO CX3CR1^GFP^ CCR2^RFP^, **d1-2)** Isolated activated microglia indicating association with thrombi + vessels. Purple arrows indicate occluded vessels, Pink asterisks show microglia near a vessel with endogenous occlusion, and yellow arrow shows leukocyte accumulation (margination and adherence). Scale bar: 50 um, panel 2nd image, 30 um.

IL-10 KO mice infected with *P. chabaudi* were used to investigate dynamics of coagulopathy and microgliosis. In the IL-10 KO CX3CR1^GFP^CCR2^RFP^ transgenic reporter mice, we could visualize and distinguish CX3CR1 + resident microglia from CCR2^+^ leukocytes, primarily monocytes and some T cells. Figure 1 presents an overview of IVM observations prefacing quantitative kinetic analyses shown in subsequent figures. Cerebral vessels are visualized by intravenous Evans blue dye (red) and Alexa 647-fibrin(ogen) (blue, Fig. 1a,b) with microglia indicated by CX3CR1-GFP (Fig. 1c,d). The IL-10 KO without fluorescent reporters was useful for visualizing fibrin(ogen) distribution relative to cerebral vasculature in the absence of fluorescent proteins GFP or RFP. Figure 1a shows a representative field in the cortex of in an uninfected IL-10 KO, in which cerebral vessels display primarily as red, with the weaker Alexa 647-fibrin(ogen) not evident. The Alexa 647- fibrin(ogen) (blue) in this field is appreciated in the supplemental video in which vessels are seen to flow (Fig. S1a). The presence of static occluding fibrin(ogen) deposits within the lumen of cerebral vessels is visible in *P. chabaudi* infected IL-10 KO mice, shown at 7 dpi in Fig. 1b (white arrow). Thrombi were easily identified, as the ‘blue’ of the fibrin(ogen) was of weak intensity when flowing but became intense when thrombi formed, due to accumulation at a localized site. Vascular occlusions of full vessel diameter, observed as dark regions devoid of flow (gold asterisk, Fig. 1b1), likely represent thrombi formed by endogenous (non-fluorescent) fibrin(ogen). IVM also clearly revealed the deposition of extravascular Alexa 647-fibrin(ogen) (blue puncta, white asterisk) or globules (white arrowhead). The larger globules were located in the proximity of cerebral vessels. Accompanying supplemental S1b-e IVM videos of the infected IL-10 KO show examples of the types of altered blood flow such as changes in speed and direction that occur during coagulopathy, and disruption to the vascular integrity in which microhemorrhages may be appreciated.

In uninfected controls, microglia had a characteristic ramified morphology with long thin processes and small soma, in which processes extended and retracted in a constant surveying pattern (Fig. 1c) In contrast, at 7 dpi infection, microglia appeared hypertrophic (shortened, thickened processes) or ameboid and often associated with vessels as shown in (Fig. 1d).

Non-fluorescent leukocytes were present as dark cells within the vasculature that ranged in diameter 7–11 µm (Fig. 1d). Supplemental movies show dynamics of these cellular and vessel elements at 7 dpi (Fig. S1c). In trials in which no vascular dye was applied, CCR2^RFP+^ cells were occasionally seen within the lumen of vessels as circular cells ranging in diameter of 10–15 µm in the uninfected control, whereas in infected animals, their numbers increased and their movement differing with many rolling and adherent cells seen, particularly in post capillary venules (Fig. S2).

### Signs of microgliosis and coagulation occur as early as post-infection day 3 in eCM

**Figure 2:**
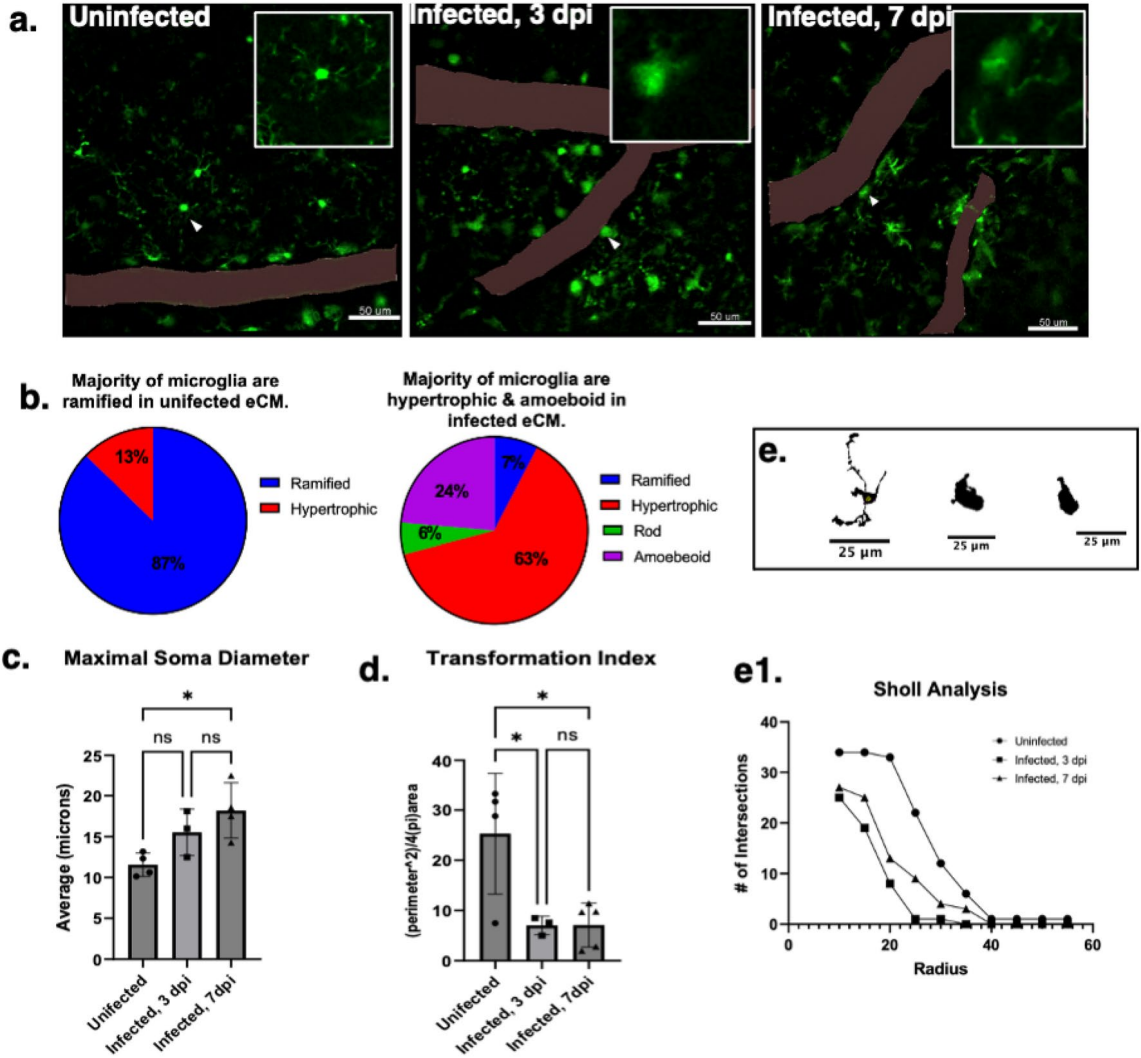
Microgliosis occurs as early as post-infection day 3 in eCM. (**a**) Max projection micrograph showing eGFP + microglia in the cortex (uninfected, infected 3 dpi, & infected 7 dpi. respectively). (**b**) Heterogeneity of microglia morphology in uninfected, 3 dpi, 7 dpi respectively. (**c**) Soma diameter of microglia increases as early as 3dpi. (**d**) Uninfected has a higher transformation index in comparison to infected 3dpi & 7 dpi. **(e)** Examples of skeletonized microglia from Sholl analysis. (**e1**) Uninfected has more intersections than infected (3 dpi & 7 dpi), more microglia processes are crossing the concentric rings. IL-10^-/-^ CX3CR1^GFP^CCR2^RFP^, *P. chabaudi*. One-way ANOVA with posthoc Holm, error bars show mean ± SD ,*p < 0.05, **p < 0.01, ***p < 0.001, ****p < 0.0001, n = 3–5 mice per group, 3–5 sites per mouse. Scale bar: 50um.

Results in Fig. 2 demonstrate that microglia become activated as early as 3 dpi. Representative micrographs show the typical morphology of microglia in uninfected, infected 3 dpi, and 7 dpi (Fig. 2a). CX3CR1^GFP^ + microglia in hyperinflammatory eCM at 7–8 dpi were classified by morphometry (ramified surveying, hypertrophic, rod-shaped, or ameboid) with most microglia found to be reactive, having hypertrophic (63%) or ameboid (24%) morphology with enlarged soma compared to uninfected controls, while in uninfected controls most microglia displayed ramified surveying morphology (Fig. 2b, 87% ramified, 13% hypertrophic). Soma size has been used as a surrogate measure of activation^24^. In infected animals, the average maximal soma diameter was 18 ± 8 µm while uninfected controls had an average diameter of 11 ± 3 µm (*P* = 0.0228) (Fig. 2c). To further show the occurrence of microglial activation, Iba-1 immunostaining was performed in non-fluorescent IL10 knockout (Fig. S3). Moreover, microglial activation and similar increase in maximal microglial soma diameter was also noted in *P. berghei* ANKA shown in supplemental (Fig. S4a,b,f), showing similarity between two separate eCM models. Microglial transformation index in uninfected controls was statistically significant higher (25 ± 12) in comparison to microglia in 3 dpi (7 ± 6) (*P* = 0.0231) and 7 dpi (7 ± 5) (*P* = 0.0178) animals, which were similar (Fig. 2d) and consistent with activation. Sholl analysis corroborated this finding of reactive microglia in infection. Microglia image masks show morphology typical of surveying ramified microglia in uninfected animals (long thin processes extending further and small soma) while typical microglia masks in infection showed hypertrophic and ameboid morphology with processes extending shorter distances (Fig. 2e-e1).

**Figure 3:**
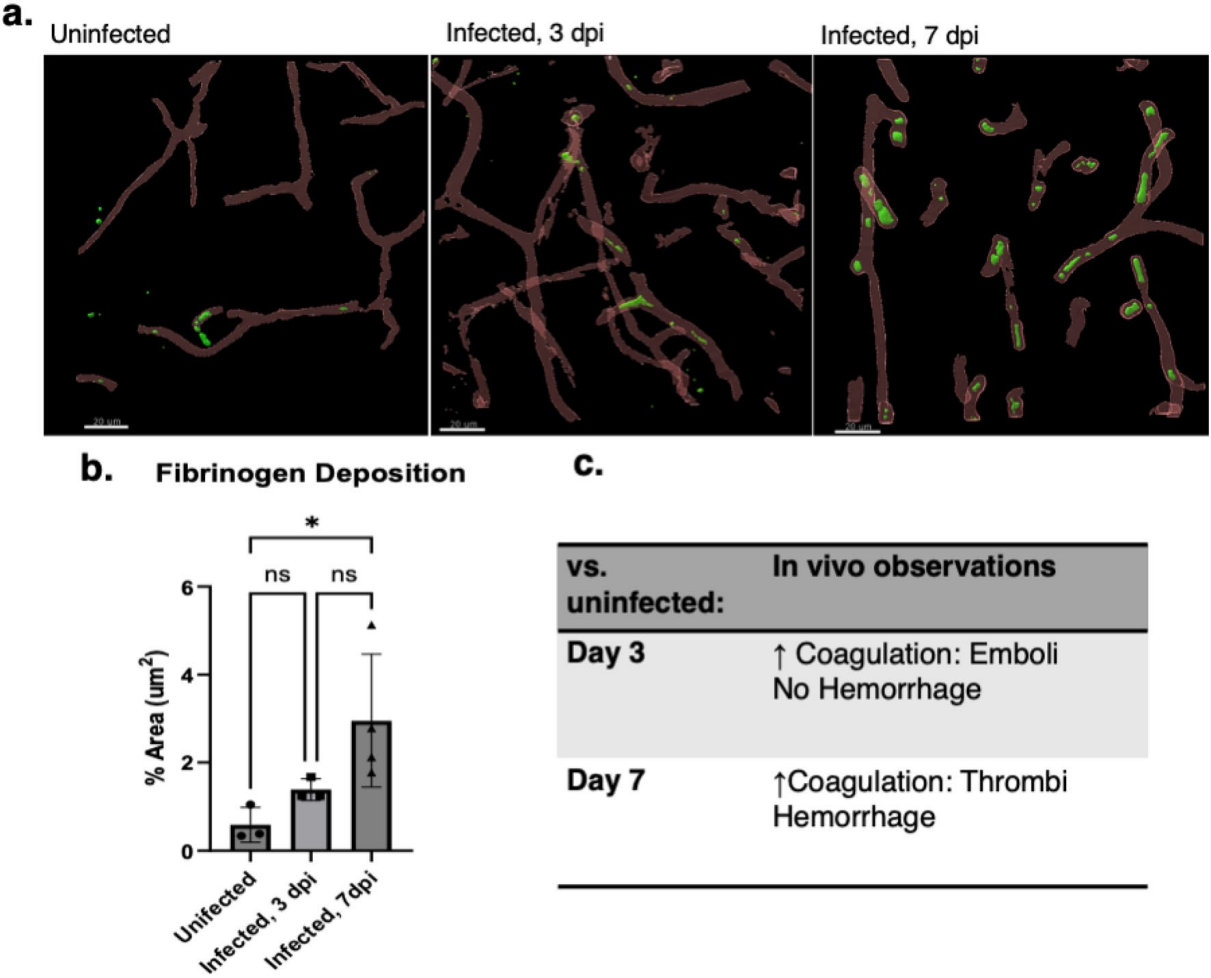
Coagulation is observed as early as day 3 and increases in severity as disease progresses. (**a**) Immunofluorescence of fibrin(ogen) in uninfected, 3 dpi, 7 dpi respectively, showing intravascular thrombi. (**b**) Enumeration of fibrin(ogen) deposition exhibiting 7 dpi with increased coagulation. (**c**) Observation of coagulopathy characteristics at day 3 and day 7 by intravital microscopy, revealing coagulation increases as disease progresses. At day 3 visual evidence of coagulation is seen as transient coagula that result as flowing coagula (emboli). By day 7 coagulation is pervasive and many static thrombi throughout vessels. Uninfected brains displayed no visual evidence of coagulation by intravital microscopy and no vessel leakage including hemorrhage. IL-10^-/-^CX3CR1^GFP^CCR2^RFP^, P. chabaudi, 7 dpi. Red lectin: Vessel and Alexa488: Fibrinogen. One-way ANOVA with posthoc error bars show mean ± SD,*p < 0.05, **p < 0.01, ***p < 0.001, ****p < 0.0001, n = 3–5 mice per group, 3–5 sites per mouse.

Fibrin(ogen) deposition was also observed early in disease via IVM time series. IF micrographs revealed increased fibrin(ogen) deposition in infected 7 dpi compared to infected 3 dpi and uninfected controls (Fig. 3a). At 7 dpi, there is a statistically significant increase in percent immunoreactive fibrin(ogen) area in the brain (2.9%) compared to uninfected controls (1.38%, 0.5%, respectively *P* = 0.0445) (Fig. 3b). IVM revealed the presence of fully occlusive thrombi in vessels at 7 dpi in contrast to 3 dpi where more mobile emboli were observed without evidence of fully occlusive vessels (Fig. 3c).

### Microglia in eCM associate strongly with cerebral microvessels

**Figure 4:**
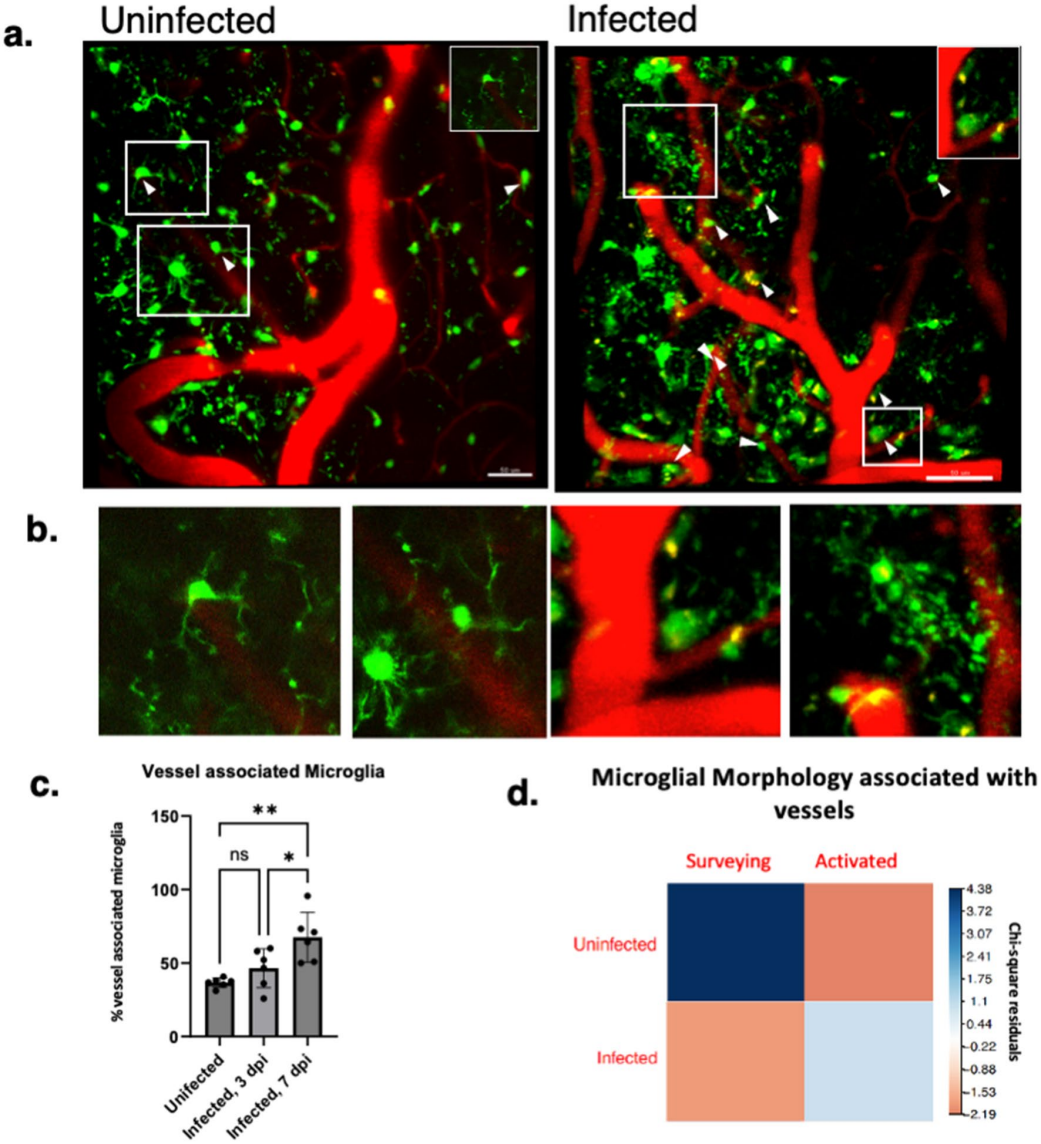
eCM results in an increase of cortical vessel associated microglia. (**a**) Max projection showing microglia aligned along vessels (i.e., vessel associated microglia), uninfected & infected 7 dpi, respectively. (**b**) Isolated microglia showing uninfected model with ramified surveying morphology and infected with ameboid morphology at vasculature. (**c**) Percent of microglia at the vasculature (vessel associated microglia) depicted as a percentage, (**d**) Association plot (Chi square analysis) correlagram representing degree of positive (blue) and negative association (orange-pink) of microglia with vessels based on morphology. Association is based on contact between microglia soma and microvessels. In infected 7dpi model reactive/activated microglia are positively associated with vessels in comparison to uninfected (positively associated with surveying microglia at the vessels). IL-10^-/-^ CX3CR1^GFP^CCR2^RFP^, *P. chabaudi*. n = 3–5 mice per group, 3–5 sites per mouse. One-way ANOVA with posthoc Holm (4**c**),Chi-square (4**d**), error bars show mean ± SD ,*p < 0.05, **p < 0.01, *** *p* < 0.001, **** *p* < 0.0001. Scale bar: 50um.

Microglia are known to associate with cerebral vasculature through chemokine dependent signaling that may increase in neuroinflammation^25^. Microglial associations with vessels in eCM were examined by quantification of vessel associated microglia (VAM) visualized by intravital microscopy. Shown in Fig. 4a are representative micrographs of uninfected and *P. chabaudi* -infected IL-10 KO CX3CR1^GFP^CCR2^RFP^ mice, respectively, indicating an increase in VAM in the infected animals versus uninfected controls. Figure 4a,b shows individual microglia at their contact points with cerebral vessels. Quantification of VAM in uninfected animals, revealed only 37 ± 3% of microglia soma made contact with vessels (soma-vessel contact), in *P. chabaudi,* while at 7 dpi (68 ± 17%) were found to make contact (*P* = 0.002), shown in Fig. 4c. An association analysis was performed to quantitively determine the association between microglia and vasculature based on activation morphology (Fig. 4d). In this visual aid, a positive value in blue indicates a positive association between microglia and vessels, while a negative value in red indicates a negative association. In the absence of infection, ramified homeostatic microglia were positively associated with vessels, while in infection reactive microglia (hypertrophic, ameboid) were more positively associated with the vasculature while ramified microglia were negatively associated with vessels (*P* = 0.001). We found that vessel association in eCM is not unique to the *P. chabaudi* model as *P. berghei ANKA*-infected mice had an increase in VAMs compared to uninfected controls (Fig. S4e).

### CCL5 is associated with fibrin(ogen) in vessels and induces microglia association to microvessels

**Figure 5:**
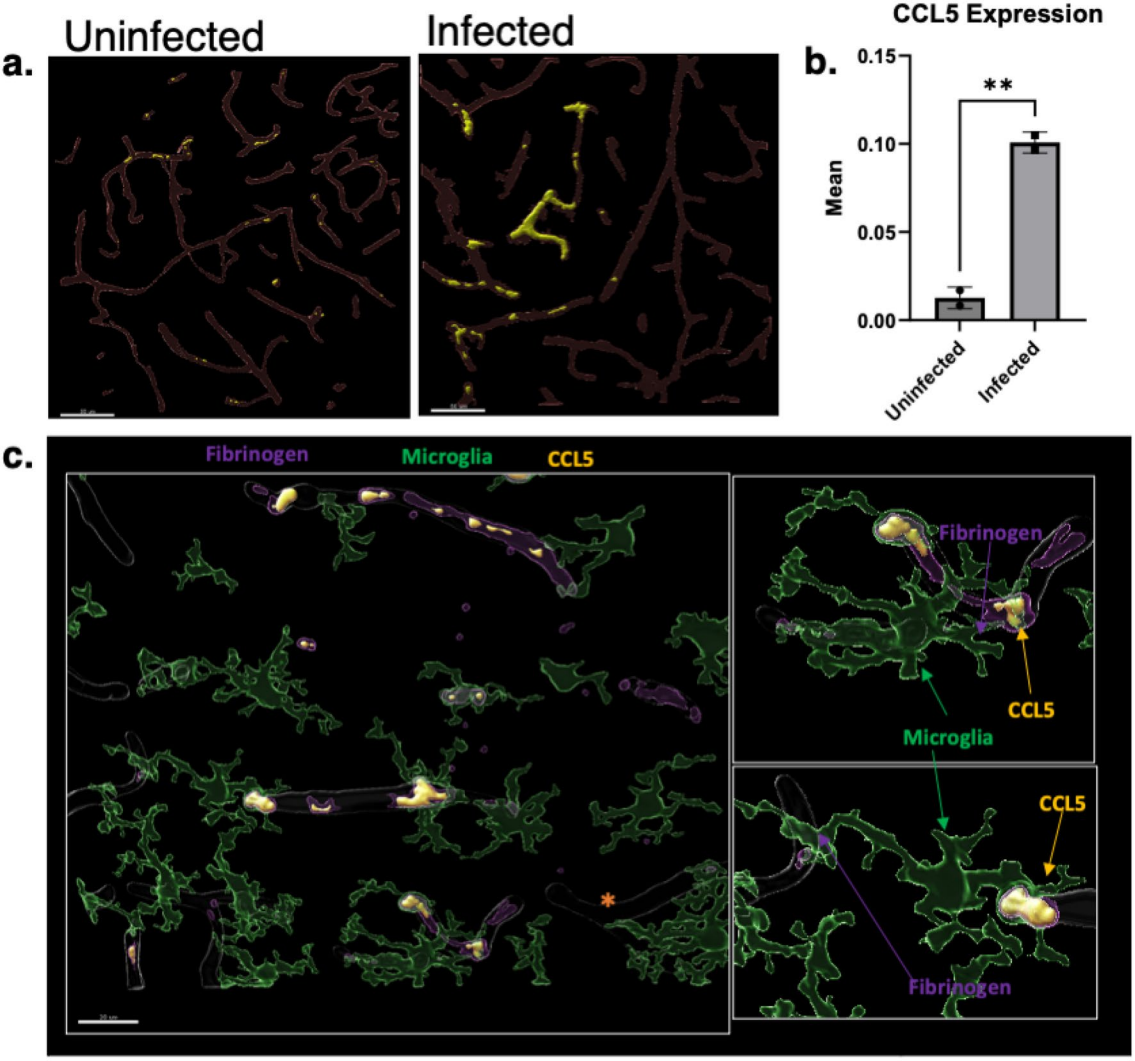
CCL5 expression in eCM is increased relative to uninfected animals. Microglia locate near foci of fibrin(ogen) thrombi and CCL5 (**a**) Micrograph of uninfected and infected murine model with + CCL5 (yellow) in the vasculature (red lectin). (**b**) Infected 7dpi has a statistically significant increase in the mean of CCL5 expression. (**c**) Volumetric reconstruction showing vasculature positive for fibrin(ogen) (purple), CCL5 (yellow) with microglia (green), isolated recons show microglia near vessels with fibrin(ogen) & CCL5. IL-10^-/-^CX3CR1^GFP^CCR2^RFP^, *P. chabaudi,* 7 dpi murine model exhibiting extravascular fibrin(ogen). Student’s t-test, error bars show mean ± SD, ,*p < 0.05, **p < 0.01, ***p < 0.001, ****p < 0.0001, n = 2 mice per group, 3 sites per mouse. Scale bar: 50um (**a**) & 20um (**c**).

C–C Motif Chemokine ligand 5 (RANTES), CCL5, a potent chemoattractant shown to attract microglia to cerebral vessels, was investigated for an increase in expression. Utilizing antibody immunofluorescence, we detected increased CCL5 expression in infected versus uninfected (Fig. 5a) brains, with the mean volume of CCL5 to be 0.10 µm^3^ in infection and 0.01 µm^3^ in uninfected brains, *P* = 0.0047 (Fig. 5b). Interestingly, the majority of CCL5 expression was localized in the vasculature and associated with areas of fibrin(ogen) deposition (Fig. 5c). In addition, microglia were also associated with these fibrin(ogen)-CCL5 regions.

### Microglia in eCM are actively engulfing fibrin(ogen)

**Figure 6:**
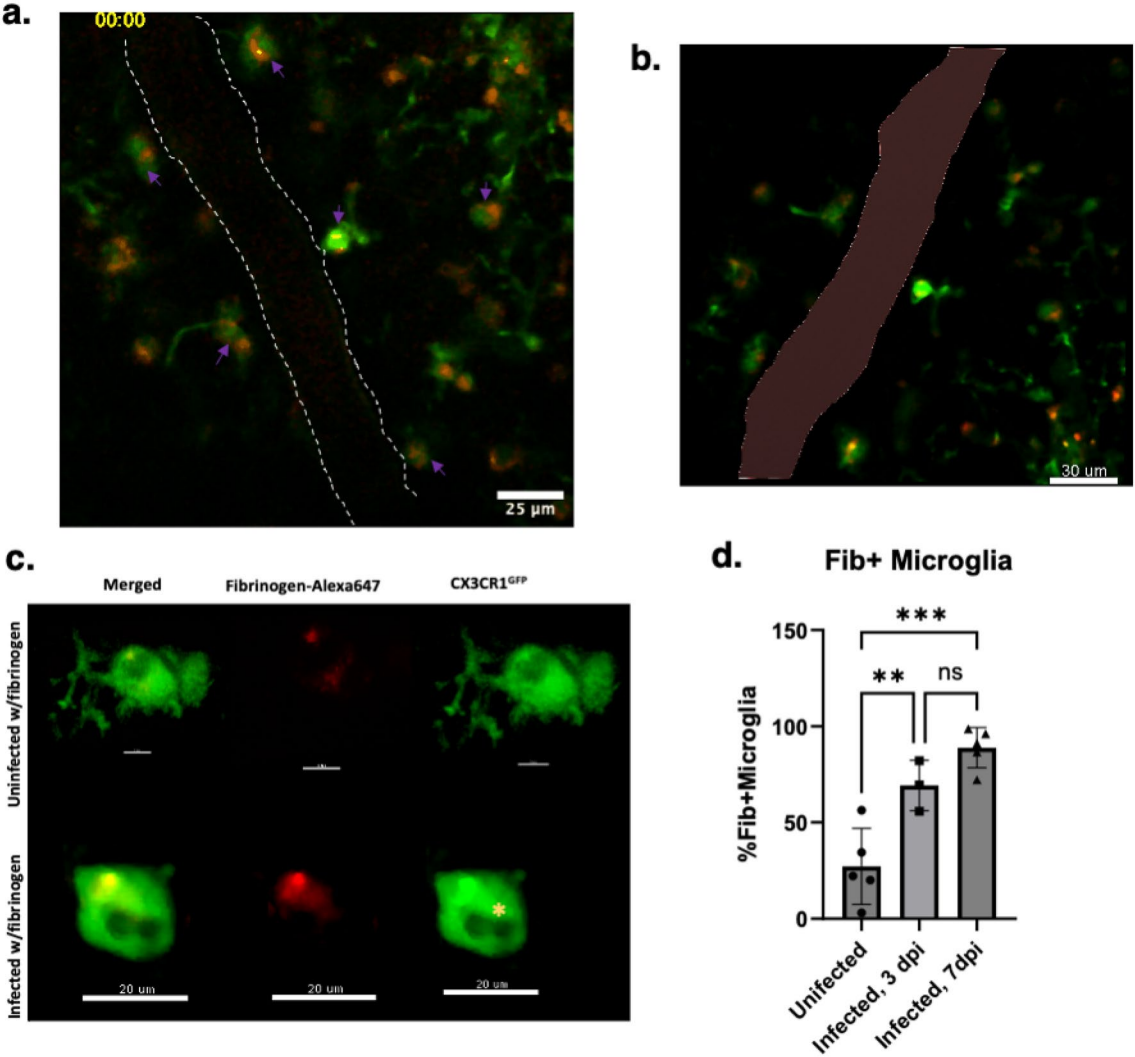
Colocalization of microglia and fibrin(ogen). (**a**) Time series showing microglia colocalized with fluorescent fibrin(ogen). (**b**) Micrograph showing microglial uptake of fibrin(ogen) Alexa647. (**c**) olumetric isolations of + GFP microglia that have phagocytosed fibrin(ogen) Alexa647, yellow star represent microglial phagosome in IL10^-/-^CX3CR1^GFP^CCR2^RFP^, *P. chabaudi* infected 7 dpi. (**d**) Percent average of fibrin(ogen) Alexa647 + microglia. IL-10^-/-^CX3CR1^GFP^CCR2^RFP^ *P. chabaudi,* 7 dpi , i.v. fibrinogen Alexa 647, One-way ANOVA with posthoc Holm, error bars show mean ± SD *p < 0.05, **p < 0.01, ***p < 0.001, ****p < 0.000, n = 3–5 mice per group, 3–5 sites per mouse.

IVM of mice treated intravenously with fluorescent fibrin(ogen) showed pervasive/persistent and dynamic uptake of fibrin(ogen) by microglia across imaged fields, visible by overlap of green CX3CR1-GFP (microglia) and red Alexa 647 fibrin(ogen) as seen in individual microglia Fig. 6a–c. Three-dimensional micrographs in Fig. 6b show the Alexa-647 fibrin(ogen) internalized in microglia that are near a vessel depicted by the reddish-brown color and micrographs also revealed that microglia appear to have a phagolysosome present in morphologically activated microglia (Fig. 6c). Quantification of the percent of microglia having an overlap with the Alexa-647 fibrin(ogen) revealed that 7–8 dpi infected mice have an average of 89% microglia with uptake of fibrin(ogen) Alexa 647 versus only 27% in uninfected mice (*P* = 0.0002), an indication that microglia in the infected model have increased interaction (phagocytosis) with fibrin(ogen) in comparison to the uninfected model. Supplemental videos reveal the dynamic interaction of microglia with fibrinogen localized within the soma (Fig. S5). Incidentally, the characteristic fibrin(ogen) deposition within and outside of cerebral vessels and fibrin(ogen)-microglial interaction seen in the *P. chabaudi* infection was observed as well in *P. berghei ANKA* infected animals as shown in supplementary materials (Fig. S4b–d).

### Microglial depletion leads to increased disease severity and hypercoagulation

**Figure 7:**
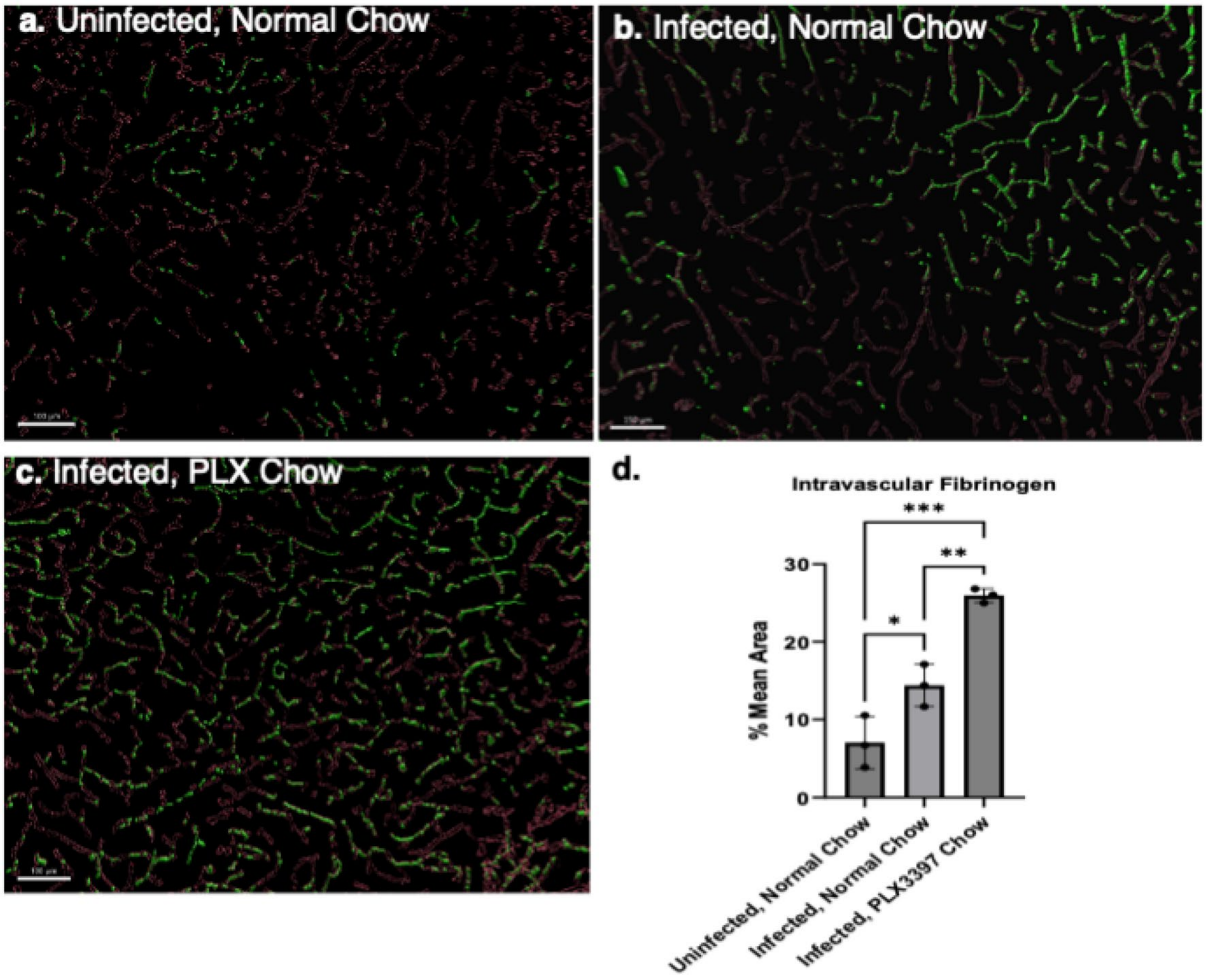
Depletion of microglia increases coagulation in eCM. Volumetric reconstructions exhibiting fibrin(ogen) (green) distribution in vessels (red) (**a**) Uninfected normal chow (**b**) Infected normal chow (**c**) Infected PLX3397 chow**,** respectively. (**d**) Percent area of intravascular fibrin(ogen). IL-10 KO, *P. chabaudi*, i.v. of fibrinogen Alexa 488, n = 3 mice per group, 3–4 sites per mouse. One-way ANOVA, posthoc Holm, error bars show mean ± SD ,*p < 0.05, **p < 0.01, *** *p* < 0.001, ****p < 0.0001. Scale bar 100 um (**a** and **c**) 150 um (**b**).

To better understand the potential role of microglia in eCM, microglia were depleted in vivo with a custom chow diet synthesized with Pexidartinib (PLX3397). Our data showed that depletion of microglia led to increased disease severity. Mice were treated with PLX3397 starting 2 weeks prior to infection of *P. chabaudi* and remained on the diet until peak of illness (7 dpi) (Fig. 7a). Microglia depletion (PLX3397 group), resulted in substantially increased coagulation throughout the brain compared to the normal chow infected group and uninfected animals (Fig. 7b–d). Quantification presented in Fig. 7d show infected PLX3397 treated mice exhibited a mean 26% area of intravascular fibrin(ogen) in comparison to infected normal chow 14% and uninfected normal chow 7% (*P* = 0.0003). Close examination of hemisected sagittal IF micrographs revealed the global distribution of fibrin(ogen) in PLX3397 treated mice to be especially concentrated in the thalamic and cortical regions of the brain.

**Figure 8:**
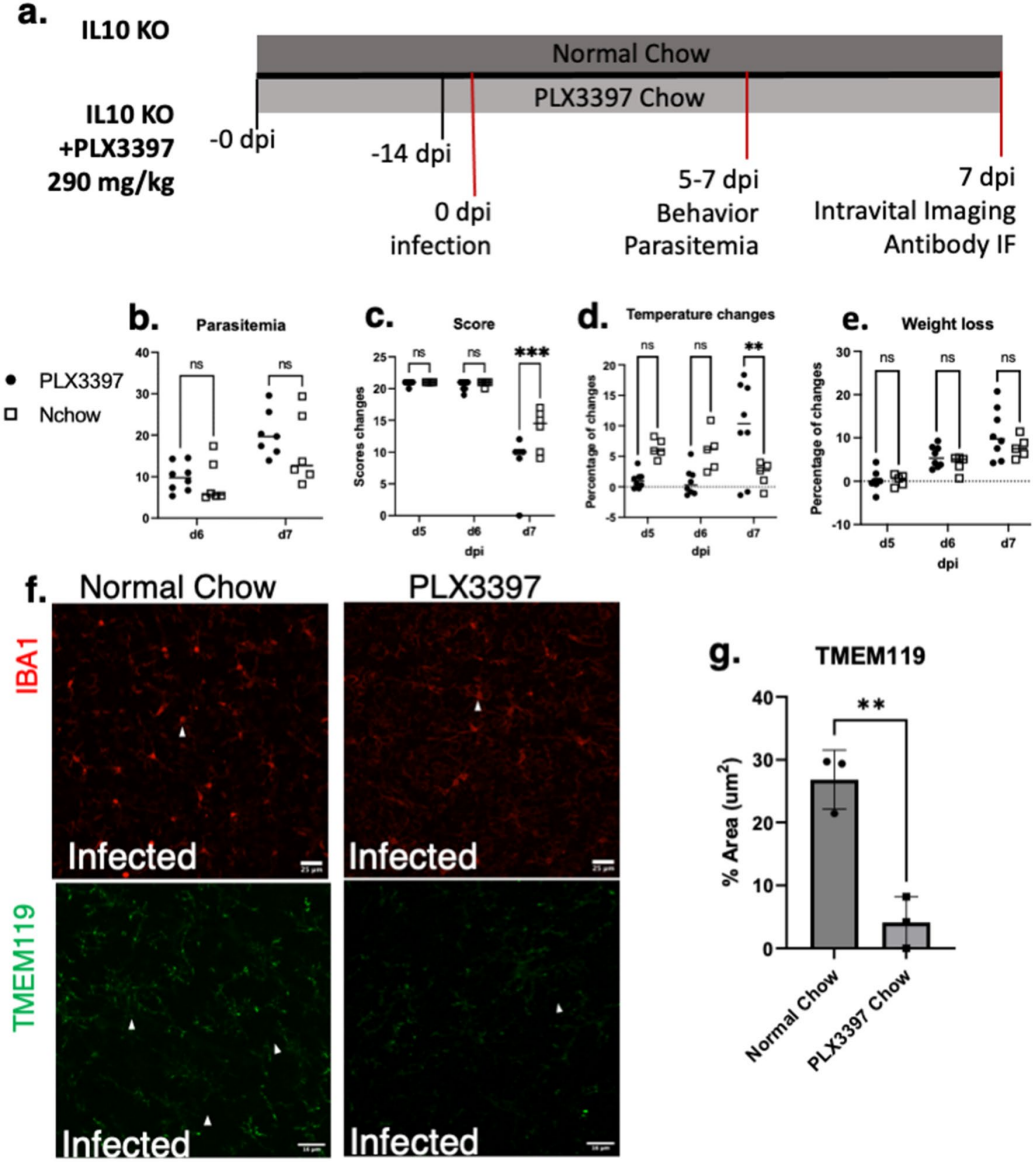
Depletion of microglia population increases severity of disease in eCM. (**a**) Schematic diagram illustrating the experimental design of PLX3397 studies. (**b**) Abbreviated SHIRPA analysis revealed a statistical difference in behavior between normal chow and PLX chow treated mice. (**c**) No significant difference in parasitemia occurred between infected mice fed the PLX chow and infected mice fed the normal chow, indicating differences are attributed to microglia depletion. (**d**) Drastic decline in temperature was noted on 7 dpi. (**e**) No statistical significance in weight loss noted. (**f**) Micrographs of Iba-1 & TMEM119 + microglia in infected mice treated with normal chow & PLX3397 chow, respectively. (**g**) % area of TMEM119 + microglia exhibiting decrease in percent area in PLX3397 chow infected group, confirming success of microglial attenuation**.** IL-10 KO, *P. chabaudi*, n = 3 with 3 sites per group. Student’s t-test, One-way ANOVA with post hoc Holm, error bars show mean ± SD,*p < 0.05, **p < 0.01, ***p < 0.001, ****p < 0.0001. Scale bar: 16um.

We observed no differences in parasitemia at the time of the peak of infection (Fig. 8a). Quantification revealed a drastic decline in the abbreviated SHIRPA screen scores of mice treated with PLX3397 between 6 and 7 dpi (Fig. 8b). We also noted, a drastic decline in temperature at 7 dpi and no changes in weight loss between normal chow infected versus PLX3397 chow infected groups (Fig. 8c,d). Microglia depletion was confirmed via immunofluorescent staining for IBA-1 and TMEM119 (Fig. 8e). Results indicated a decrease in percent area of TMEM119 in infected-PLX3397 treated groups in comparison to infected-normal chow groups (4.12 ± 3% vs 26.86 ± 5%, respectively *P* = 0.0032), confirming decrease of microglia population. Supplementary materials show IVM observations in which infected-PLX3397 treated mice exhibited coagulation, microhemorrhages evident in movies (Fig. S6) and leukocyte accumulation. Additionally, in comparison of intravascular and extravascular fibrin(ogen) deposition in ex vivo sections, increases occurred with PLX3397 treatment in both the intravascular component, which was the majority component, and the extravascular component (Fig. S7a,b). Fig. S7c shows flow cytometry results of splenic cells indicating that compared to normal chow fed mice, PLX3397 mice had no significant changes in CD11b + F4-80 + (macrophages) or Ly6c + (monocytes).

## Discussion

As CNS-resident immune cells, microglia dynamically survey and respond to stimuli in their microenvironment to maintain homeostasis. Though there is indication of microglial activation in human CM and eCM, there remain unknowns regarding the temporal and dynamic interactions with cerebral vessels including responses to coagulopathy in eCM. In this work, we applied IVM and immunofluorescence to study microglia responses in eCM progression with severity. We show that microglia display a reactive morphology early in disease in *P. chabaudi* infection, are highly associated with the vasculature, respond to intravascular coagulation events, and actively uptake fibrin(ogen). When depleted, disease severity and coagulopathy both increase, implying microglia play a critical role in ameliorating coagulopathy in eCM. Complementary studies in *P. berghei ANKA* exhibited similarity in vessel occlusions, microglial activation, and increases in vessel-associated microglia, indicating these observed findings are conserved across different eCM models, an important point as *P. berghei ANKA* is the most commonly studied eCM model.

IVM provided real-time dynamic visualization of coagulation and the role microglia may be playing in eCM (Fig. 1, supplement movies 1b, d). Image stacks revealed microglia as having a reactive morphology identified by CX3CR1-GFP^+^ CCR2-RFP^-^ expression in the IL-10 KO CX3CR1^GFP^CCR2^RFP^ model and confirmed by postmortem IBA1 staining. Notably, microglia are activated as early as 3 dpi in infection, seen by IVM and confirmed quantitively through metrics for assessment reactivity (Fig. 2). Further, results suggests that microglial activation occurs before neurological decline is present in mice. This finding of early brain microglia activation is consistent with findings in murine ANKA, in which early activation of microglia near vessels through ex vivo techniques in the retina of fatal murine cerebral malaria was reported^26^. Along with microglial activation, our studies showed coagulation also begins early in infection at 3 dpi, were we visualized formation of emboli in vivo and actively undergo fibrinolysis (Fig. 3). Subsequent ex vivo immunofluorescence showed little evidence of coagulation, likely a consequence of perfusion eliminating slow moving/loosely anchored clots. IVM thus was critical in revealing increase in coagulation with dynamic maintenance at the cerebral vessel level. At 7 dpi, cortical vasculature had fully occlusive thrombi lodged in vessels that resulted in blocked perfusion throughout fields imaged in vivo, seen as widespread fibrin(ogen) deposition in ex vivo immunofluorescence. Our findings indicate a dynamically changing progression in vessel occlusion that appears to be due to dysregulated coagulation as disease progresses, which results in the inability of the coagulum to breakdown at advanced disease. Coagulation has been previously shown to occur in eCM through plasma indicators of the c protein anticoagulation pathway^27^. Data presented in this work did not investigate if fibrinogen levels varied throughout the entirety of infection or if modulated in the course of disease due to compensatory fibrinolysis and has been alluded to occur in humans with CM^28^. Increased fibrinogen was reported to occur in the liver at post infection day 7 compared to uninfected controls in this model, a potential indication of increased production overall^15^. Future studies will need to examine liver and brain fibrin(ogen) levels over time to better understand the elements of fibrin(ogen) increases and vessel thrombosis reported in this report.

Microglia were highly associated with the vasculature in this infection model, reported in Fig. 4 as vessel associated microglia (VAM), an association that was most pronounced at 7 dpi. Notably, increase in VAM was found in *P. berghei ANKA* as well. In *P. chabaudi* infection, activated/reactive microglia were positively associated with the cerebral vasculature, (Fig. 4d) suggesting recruitment of microglia to cerebral vessels, which has been previously reported^25^. In one study, CCL5 (RANTES), a potent chemoattractant for immune cells such as blood monocytes and memory T-helper cells, was mechanistically shown to drive microglial movement to cerebral vessels^25^. In the current study, the expression of CCL5 in the brain, examined by IF, increased substantially versus uninfected mice, with volumetric renderings showing CCL5 localized to the cerebral vasculature and, more specifically, to occlusive fibrin(ogen)-positive thrombi (Fig. 5). A striking observation was that microglia were observed next to the vasculature at sites exhibiting fibrin(ogen)-labeled thrombi with CCL5, suggestive that microglia were attracted to CCL5 expressing cells. CCL5 was localized within the coagula as spherical shapes of size consistent with leukocytes (10–14 µm), suggesting, immune cells trapped within the thrombi may be playing a role in CCL5 production to attract microglia. Platelets, a critical component of thrombi, secrete chemokines (including CCL5),to initiate or promote inflammatory responses at the site of vascular injury–responses that include recruitment of inflammatory mediating cells to the vascular wall^29,30^. Microglia highly express CCR5, a receptor for which CCL5 serves as a ligand. This study indicates that in eCM CCL5 is a likely chemoattractant that signals microglia to the cerebral vessels. Previous studies have indicated that in humans CM CCL5 (RANTES) expression level was linked to patient mortality, such that patients who succumbed to disease exhibited low levels of RANTES^29^. Thus, in future studies, it will be of interest to investigate the use of a CCL5 agonist to confirm the role of CCL5 in attracting microglia and determine if the recruitment of microglia to the vasculature is a necessary element for these immune cells to respond to coagulation events. Additional studies should examine if CCL3 (MIP1-alpha) or CCL4 (MIP1-beta) could impact microglia recruitment in eCM, as well.

IVM and quantitative analyses in this study also show that not only are microglia highly associated with cerebral vasculature, but they are also actively interacting with coagulation elements. Fibrin(ogen) was deposited in the intravascular space as well in the perivascular space either co-localized with microglia or as small, scattered puncta beyond these cells. A significant increase in the number of Fibrin(ogen)^+^ microglia (Alexa647-fibrin(ogen)) in *P. chabaudi* infection brains relative to uninfected controls occurred (Fig. 6d), with microglia containing round phagosome-like structures (Fig. 6c). The imaging objective used in these studies provided a lateral resolution of approximately 0.44 µm and 2.3 µm axial resolution, providing sufficient resolution to visualize microglial phagosomes which range in size from ~ 0.5 −7 µm; microglia phagosome fusion is an indicator of active phagocytosis. A non-reporter mouse infected with *P. chabaudi* and given i.v. fluorescent fibrinogen, confirmed the in vivo distribution of extravascular fluorescent fibrin(ogen) as puncta scattered throughout perivascular space in the absence of GFP or RFP fluorescence (white arrows of Fig. 1b, movie in Fig. S1b-d) and also showed it contained in cell-like clusters, presumably (unlabeled) microglia, given they moved in manner similar to surveying microglia in the CX3CR1-GFP models (providing an interesting manner to identify these cells). The perivascular distribution of fibrin(ogen) likely resulted from compromised BBB as leakage and microhemorrhage were noted features as in other eCM models (Fig. 1). The uptake of fibrin(ogen) by microglia was evident though it was difficult to determine degree of uptake of extravascular fibrin(ogen) vs. intravascular, the latter believed to occur because uninfected animals that did not have leaky vessels also displayed fibrin(ogen) uptake by microglia in contact with vessels (Fig. 6c,d) and because processes appeared at times to cross into the vessel wall (Fig. S4g). This is consistent with studies indicating contact of microglia processes with endothelium^25^. Indication that microglia were also uptaking extravascular fibrin(ogen) came from Fig. S7 data showing that with infection there wasn’t a statistically significant increase in extravascular deposits though IVM showed extravasated fibrin(ogen) occurred, but did increase when microglia were depleted. It would be of interest to explore the nature of cell-dependent processing of fibrin(ogen) in the future given there are multiple mechanisms in which immune cells promote fibrinogen clearance^31^. In neurodegenerative models, microglia interact with fibrinogen in a CD11b/CD18 dependent manner, promoting phagocytosis and microglial activation^19,20, 32^ and has not yet been examined in eCM.

PLX3397 treatment resulted in increased disease severity, with mice presenting with a rapid decline in neurological scores and exacerbated hypothermia compared to the normal chow infection group on 7 dpi (Fig. 7). It cannot be assumed all effects observed are attributed to reduced microglial population. Although CSF-1R inhibitors have in some cases been shown to deplete other immune cell populations, Szalay et al. showed that 3 weeks PLX3397 treatment did not result in splenic macrophage depletion or in CD4 + and CD8 + T lymphocytes, CD19 + MHCII + B cells, CD11b + Ly6c^high^ monocytes, CD11b^low^ Ly6c^high^ cells, and CD11b + Ly6c^low^ cells in comparison to normal chow uninfected mice^33^. In this study, flow cytometry indicated that PLX3397 treatment did not deplete splenic CD11b + F4-80 + (macrophages) or Ly6c + (monocytes) in infected groups (Fig. S7c), providing indication that microglial depletion is a major contributor of observed effects. Effects on other organs should be considered- while liver fibrinogen increases in *P. chabaudi* infection^15^, we do not know the direct effects of PLX3397. Finally, there may be a complex interplay between temperature and coagulation in the brain that warrants consideration. Hypothermia can inhibit the ability for coagulation to occur, however once coagulation occurs and clots are formed, hypothermia has no effect on clotting^34^. Neurogenic temperature regulation is complex though brain injury is known to lead to dysregulation, the nature of this dysregulation in eCM should be further studied. It is clear that in these studies there is pervasive vessels occlusion and stasis throughout the brain that is further exacerbated by microglial depletion impacting the severity of disease and likely viability.

These studies showed when microglia are depleted there is an increase in fibrin(ogen) deposition (Fig. 8) throughout the brain. The thalamus and cortex exhibited the highest increase in deposition. These brain regions are impacted in human CM, exhibiting vessel blockage (i.e. occlusion) and preventing perfusion. Most patients who survive CM face neurological sequelae which include behavioral and cognitive deficits, functions in which the thalamus and cortex play a role^2,34^. The thalamus is responsible for relaying information between the cortex & brain stem and plays a role in sleep, wakefulness, conscience, learning, and memory. While the cortex is responsible for motor, language, higher-level processing, memory, thought, emotion, and reasoning^35^. Many of these functions are negatively impacted in patients presenting with neurological sequelae^2^. These regions should be further investigated for neuropathological features and their potential role in behavioral/neurological dysfunction observed in and beyond infection.

Noting the increase in hypercoagulation in the PLX3397 depleted-microglia group, we hypothesize that microglia play a role in uptake and processing fibrin(ogen) resulting from increased coagulation during infection. Fluorescent fibrin(ogen) was observed in the extravascular space, extravasating with microhemorrhages and could have been one source of fibrin(ogen) phagocytized. An intriguing possibility is that microglia could also uptake fibrin(ogen) from the vasculature. In one study, CCL5-mediated vessel associated microglia expressed Claudin 5 allowing processes to come into direct contact with endothelial cells^25^. The current study showed an increase in vessel associated microglia and CCL5 expression (Fig. 5b). Furthermore, intravital microscopy revealed microglia processes interacting with the vasculature, potentially entering the luminal space (Fig. S4g). Thus, we hypothesize another mechanism is at play in which microglial processes interact with the endothelium and secrete tissue plasminogen activator (tPA) to aide in fibrin degradation and uptake fibrinogen^36^. This possibility that microglia uptake contents from cerebral vessels is supported by observations of a small amount of uptake of either Evans blue or fluorescent fibrin(ogen) found only in vessels in uninfected controls in which no evidence of leaky vessels was found (Fig. S1a). In infection, where vessel leakage was evident, fibrin(ogen) was found in the perivascular space. It is not clear what portion of fibrin(ogen) taken up by microglia was from vessels or parenchyma. This aspect, along with the nature of the interaction, will be the focus of future studies.

While studies are needed to closely examine the interaction of microglia with fibrin(ogen) the studies presented in this current work, strongly suggest that, in eCM, microglia can detect vascular abnormalities such as hypercoagulation and interact with the vessels to aid in fibrinolysis.

These studies highlight a potentially critical protective function of microglia in eCM, via their recruitment to the cerebral vasculature in infection. It has already been reported that children with CM having lowered levels of CCL5 (RANTES) were more likely to die from this complication of severe malaria than children with higher levels of RANTES. If in fact RANTES is responsible, even in part, for recruitment of VAMs in infection, it is conceivable, given our study findings, that the observed differences in survival could be related to not only how microglia function to combat coagulopathy and function to maintain homeostasis, but also to the ability to recruit these immune effector cells to the vasculature, which serves as a central intersecting hub for coagulopathy and neuroinflammation. In the future, it will be of interest to employ intravital microscopy to study in vivo responses in mice of younger age than the standard 6–12 weeks of age (young adult) used in eCM to give insights into why CM is more lethal in children than adults^37^, and which may be possible given new research establishing *P. chabaudi* infection in 15 day old pups^37^.

### Supplementary Information

Supplementary Information.

## Data Availability

All relevant data is available from the corresponding author upon request.
